# Cytoprotective Activity of Polyamines Is Associated with the Alternative Splicing of *RAD51A* Pre-mRNA in Normal Human CD4^+^ T Lymphocytes

**DOI:** 10.3390/ijms23031863

**Published:** 2022-02-07

**Authors:** Yulia A. Gladilina, Lylia Bey, Abdullah Hilal, Ekaterina V. Neborak, Varvara G. Blinova, Dmitry D. Zhdanov

**Affiliations:** 1 Institute of Biomedical Chemistry, Pogodinskaya St. 10/8, 119121 Moscow, Russia; leonova_y@mail.ru (Y.A.G.); hilalabdullahh@gmail.com (A.H.); varya.blinova@list.ru (V.G.B.); 2 Department of Biochemistry, Peoples’ Friendship University of Russia (RUDN University), Miklukho—Maklaya St. 6, 117198 Moscow, Russia; beylylia60@gmail.com (L.B.); neborak-ev@rudn.ru (E.V.N.)

**Keywords:** polyamines, alternative splicing, DNA damage, cytoprotection, apoptosis

## Abstract

Physiological polyamines are ubiquitous polycations with pleiotropic biochemical activities, including regulation of gene expression and cell proliferation as well as modulation of cell signaling. They can also decrease DNA damage and promote cell survival. In the present study, we demonstrated that polyamines have cytoprotective effects on normal human CD4^+^ T lymphocytes but not on cancer Jurkat or K562 cells. Pretreatment of lymphocytes with polyamines resulted in a significant reduction in cells with DNA damage induced by doxorubicin, cisplatin, or irinotecan, leading to an increase in cell survival and viability. The induction of *RAD51A* expression was in response to DNA damage in both cancer and normal cells. However, in normal cells, putrescin pretreatment resulted in alternative splicing of *RAD51A* and the switch of the predominant expression from the splice variant with the deletion of exon 4 to the full-length variant. Induction of *RAD51A* alternative splicing by splice-switching oligonucleotides resulted in a decrease in DNA damage and cell protection against cisplatin-induced apoptosis. The results of this study suggest that the cytoprotective activity of polyamines is associated with the alternative splicing of *RAD51A* pre-mRNA in normal human CD4^+^ T lymphocytes. The difference in the sensitivity of normal and cancer cells to polyamines may become the basis for the use of these compounds to protect normal lymphocytes during lymphoblastic chemotherapy.

## 1. Introduction

Polyamines (PAs) are small polycationic molecules derived from the metabolism of ornithine by the enzyme ornithine decarboxylase [1]. Spermine (Spm), spermidine (Spd), and putrescine (Put) are three main PAs found in all types of human cells and tissues [2]. Due to their positive charge, PAs are able to bind most biological polymers. The binding of PAs to proteins results in modulation of the activity of different enzymes [3] and ion channels [4] that support the functions of cell membranes. Due to their ability to interact with nucleic acids, PAs are able to influence gene transcription [5,6] and mRNA translation [7,8]. Thus, PAs are essential for different cellular functions, including cell growth and proliferation [9]. Several works have demonstrated that PAs have antioxidative effects [10], can promote homology-directed DNA repair [11], and downregulate DNA damage-associated cell death [12,13]. Almost all cells can produce PAs, but their production is especially high in rapidly growing cells; therefore, the concentration of PAs as well as the gene expression and activity of enzymes involved in PA biosynthesis are higher in cancer tissues than in normal surrounding tissues [14,15,16]. Therefore, the pathway of PA metabolism is a promising target for chemotherapy and chemoprevention [17]. PAs have roles in normal immune cell function [18] and are responsible for T cell proliferation and differentiation [19]. Thus, an anti-PA chemotherapeutic strategy may have a negative effect on normal immune cells. To support this, catabolic products of PA oxidation are toxic and can induce apoptosis in normal and tumor cells [20,21,22,23]. Several works have demonstrated that the ability of PAs to induce a cellular response to DNA damage is associated with the RAD51 DNA repair system [11,24]. To date, there are no works comparing the ability of PAs to influence DNA damage and the survival of cancer and normal cells. In the present study, we demonstrated that PAs have cytoprotective effects on normal human CD4^+^ T lymphocytes but not on Jurkat or K562 cancer cells, and such protection is associated with the induction of the alternative splicing of *RAD51* pre-mRNA.

## 2. Results

### 2.1. Polyamines Have Cytoprotective Activity against Normal CD4^+^ T Cells but Not against Malignant Cells

**Figure 1:**
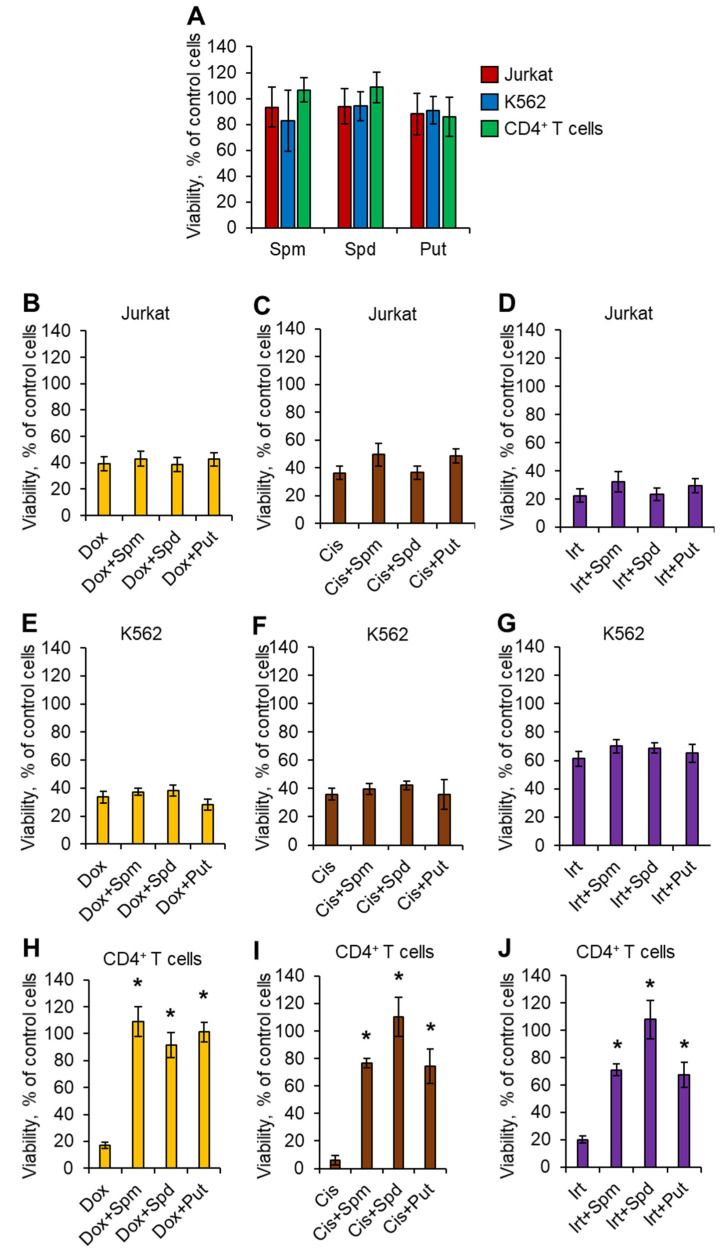
Cytoprotective activity of PAs in normal CD4^+^ T cells. (**A**) Results of the MTT test for cells incubated with 10 µM of each PA for 72 h. The results of the MTT test for cancer (**B**–**D**) Jurkat, (**E**–**G**) K562, or (**H**–**J**) normal CD4^+^ T cells incubated with genotoxic agents: 1 µM doxorubicin (Dox), 0.5 µM cisplatin (Cis), or 1 µM irinotecan (Irt) in the presence or absence of each PA. *n* = 8. * *p* ≤ 0.05 vs. cells not treated with PA. Spd: spermidine; Spm: spermine; Put: putrescine.

It is known that PAs can suppress or induce immune cell growth depending on their concentration in cell media [25,26]. We tested the ability of Spm, Spd, or Put to affect cell growth at a concentration of 10 µM using the MTT test. The results are shown in Figure S1A,B in the Supplementary File. All three PAs at this concentration did not show significant activity to induce or suppress the growth of either Jurkat or K562 cancer cells or normal activated CD4^+^ T lymphocytes (Figure 1A). The results of the MTT test demonstrating the dose-dependent reduction in cell metabolic activity/growth after incubation with DNA-damaging agents are shown in Figure S1D–F in the Supplementary File. The incubation of cancer cells with DNA-damaging agents resulted in significant inhibition of their metabolic activity/growth up to 32.4–68.3% of control cells, and the addition of each PA did not protect cells against damage (Figure 1B–G). CD4^+^ T cells were more sensitive to DNA damage, and only 3.1–20.9% of cells remained alive after incubation with genotoxic agents (Figure 1H–J). However, pretreatment of CD4^+^ T cells with PAs resulted in a significant induction of cell metabolic activity/cell growth up to 55.3–122.6% of control nontreated cells. Representative photos of the MTT assay for these experiments are presented in Figure S2 in the Supplementary File. These results demonstrated that each PA has a cytoprotective effect on normal but not cancer cells. The simplest PA is Put, which originates from ornithine and can be successively converted to both Spd and Spm [27]. Thus, all subsequent experiments were performed with Put as a cytoprotective agent.

### 2.2. Polyamines Decrease DNA Damage Induced by Genotoxic Agents in Normal CD4^+^ T Cells

**Figure 2:**
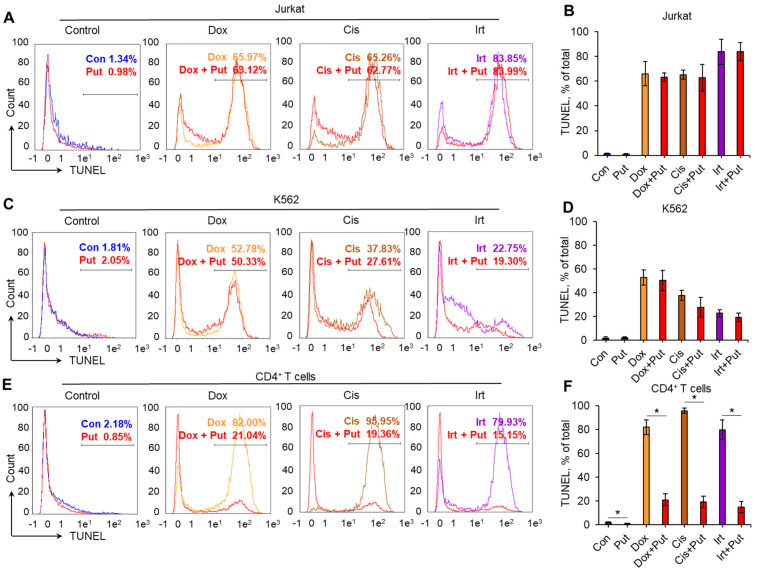
Decreased DNA damage in normal CD4^+^ T cells pretreated with PAs. Representative terminal deoxynucleotidyl transferase-mediated d-UTP nick end labeling (TUNEL) flow-cytometry diagrams for cancer cell lines (**A**) Jurkat, (**C**) K562, or (**E**) normal CD4^+^ T lymphocytes pretreated with each PA and incubated with genotoxic agents. The results of the TUNEL assay for flow cytometry for treated (**B**) Jurkat, (**D**) K562, or (**F**) CD4^+^ T cells. *n* = 4. * *p* ≤ 0.05. Con: control intact cells; Cis: cisplatin; Dox: doxorubicin; Irt: irinotecan; Spd: spermidine; Spm: spermine; Put: putrescine. *n* = 4. * *p* ≤ 0.05.

We studied whether Put can decrease the level of DNA damage and performed a TUNEL assay for flow cytometry. Put was not able to significantly reduce the proportion of cells treated with each DNA-damaging agent (Figure 2A–D). However, a significant reduction in TUNEL-positive cells was observed for CD4^+^ T lymphocytes pretreated with Put: by 61.1% for Dox, 76.6% for Cis, and 64.8% for Irt (Figure 2E,F). The results of this experiment demonstrated that Put induced significant downregulation of DNA damage in normal CD4^+^ T cells but not in cancer cells. Among the studied cytotoxic agents, Cis demonstrated the strongest activity to induce DNA damage (up to 96.3%), and such an induction was successfully diminished by Put. Consequently, experiments were performed with Cis as a DNA-damaging agent.

### 2.3. Putrescin Prevents the Progression of Apoptosis Induced by Cisplatin in Normal CD4^+^ T Cells

**Figure 3:**
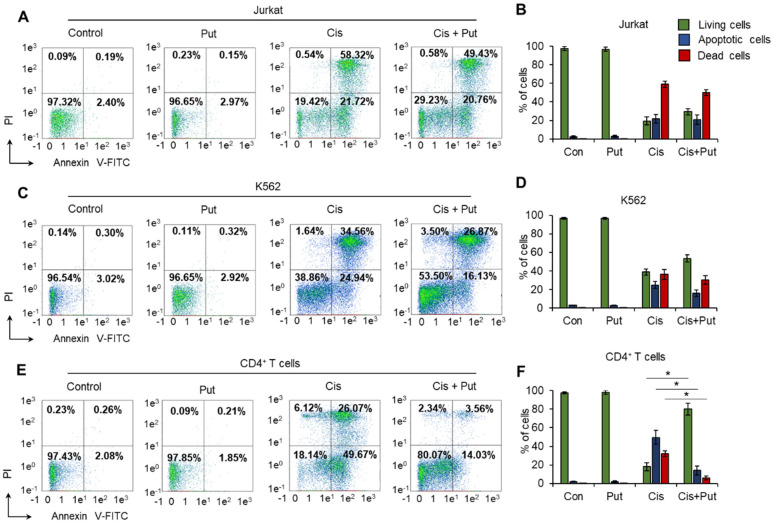
Prevention of cisplatin-induced apoptosis by putrescin (Put) in normal CD4^+^ T cells. Cells were labeled with annexin V-FITC and propidium iodide, and flow cytometry was performed 72 h after incubation with cisplatin (Cis). Representative plots for cancer cell lines (**A**) Jurkat, (**C**) K562, or (**E**) normal CD4^+^ T lymphocytes pretreated with Put and incubated with Cis. The proportions of live cells (lower left quadrants), apoptotic cells (lower right quadrants), and dead cells (two upper quadrants) are presented. (**B**,**D**,**F**) Histograms of live, apoptotic, and dead cells measured by flow cytometry. *n* = 4. * *p* ≤ 0.05.

The results of the MTT test do not allow us to answer whether Put prevents cell death, as this assay determines the metabolic status of cells. We measured the induction of cell death by Cis in Put-treated and nontreated cells by labeling phosphatidyl serine on apoptotic cell membranes with annexin V-FITC and cell DNA by PI followed by flow cytometry. The results of the cell death measurement were in good agreement with the results from the MTT test (Figure 1) and with the induction of DNA damage determined by the TUNEL assay (Figure 2). A concentration of 0.5 µM Cis could induce apoptotic cell death. CD4^+^ T lymphocytes and Jurkat cells were the most sensitive (17.3–21.4% of them remained alive), while K562 cells were more resistant (34.1–39.6% remained alive) (Figure 3). Pretreatment of cells with Put led to a small and insignificant decrease in the proportion of apoptotic and dead Jurkat (Figure 3A,B) and K562 (Figure 3C,D) cell lines. However, pretreatment of normal CD4^+^ T cells with Put resulted in significant prevention of cell death, and more than 80% of cells remained alive after the induction of apoptosis with Cis (Figure 3E,F). The results of this experiment indicate that PAs can prevent cell death associated with DNA damage in normal lymphocytes but not in cancer lymphocytes.

### 2.4. Cisplatin Induces RAD51A but No Other RAD51 Family Members in Cancer Cell Lines and Normal CD4^+^ T Cells

**Figure 4:**
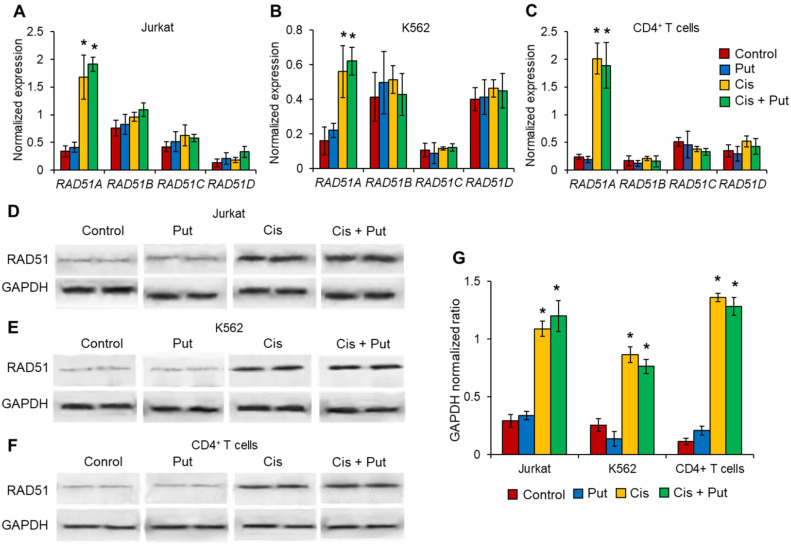
Induced RAD51A expression in cells treated with cisplatin (Cis). mRNA levels of *RAD51* members measured by real-time RT-PCR in cancer cell lines (**A**) Jurkat, (**B**) K562, or (**C**) normal CD4^+^ T lymphocytes pretreated with putrescin (Put) and incubated with Cis. mRNA levels were normalized relative to the expression of the reference gene *18S*. (**D**–**F**) Western blotting for RAD51 protein and the reference protein GAPDH in treated cells. (**G**) Results of RAD51 protein quantification relative to GAPDH. *n* = 4. * *p* ≤ 0.05 vs. control intact nontreated cells.

PAs are able to facilitate homologous recombination-mediated DNA repair through the modulation of RAD51 activity [11,24]. Four genes encoding RAD51 paralogs are known in the human genome: *RAD51A*, *RAD51B*, *RAD51C*, and *RAD51D* [28]. We investigated the expression levels of each *RAD51* family member by real-time RT-PCR in cancer and normal lymphocytes pretreated with Put and incubated with Cis. We observed that Cis could induce the expression of only *RAD51A* in cancer and normal cells (Figure 4A–C). Put did not modulate *RAD51A* expression. The mRNA levels of other *RAD51* members, *RAD51B*, *RAD51C*, or *RAD51D*, were unchanged in all cell types treated with Cis and/or Put. Western blotting results of total RAD51 protein were in agreement with the results from real-time RT-PCR (Figure 4D–G). We observed the induction of RAD51 protein levels in all types of cells treated with Cis.

### 2.5. Putrescin Induces Alternative Splicing of RAD51A Pre-mRNA in Normal CD4^+^ T Cells but Not in Malignant Cells

**Figure 5:**
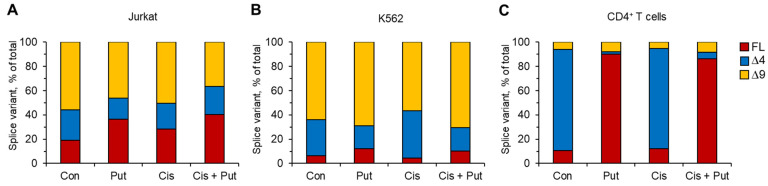
Induction of alternative splicing of *RAD51A* pre-mRNA by Put. mRNA levels of RAD51 splice variants measured by real-time RT-PCR in cancer cell lines (**A**) Jurkat, (**B**) K562, or (**C**) normal CD4^+^ T lymphocytes pretreated with Put and incubated with cisplatin (Cis). mRNA levels of splice variants were normalized relative to the expression of the reference gene *18S*. *n* = 4. FL: full-length splice variant. ∆4: mRNA splice variant with the deletion of exon 4. ∆9: mRNA splice variant with the deletion of exon 9.

The results of previous experiments did not allow us to answer why Put prevented the death of normal CD4^+^ T cells but not cancer Jurkat or K562 cells. The pre-mRNA of *RAD51A* is subjected to alternative splicing leading to the induction of three main splice variants: full-length (FL) and truncated splice variants with deletion of exon 4 (∆4 splice variant) or exon 9 (∆9 splice variant) in mature mRNA transcripts. We measured the proportion of mRNA of each *RAD51* splice variant in cells pretreated with Put and incubated with Cis by real-time RT-PCR. We observed that neither Put nor Cis was able to significantly shift the proportion of spliced mRNA in both cancer cell lines (Figure 5A,B). In both of these cells, the ∆9 variant was the predominant variant. The ∆4 splice variant and FL variant were minor in Jurkat and K562 cells, respectively. In normal nontreated CD4^+^ T lymphocytes, the predominant variant was ∆4 (approximately 79.6% of total *RAD51A* mRNA), and its proportion did not change after incubation with Cis (Figure 5C) regardless of total *RAD51A* induction in response to Cis (shown in Figure 4C). We observed significant induction of the proportion of FL variants in CD4^+^ T cells treated with Put. The proportion of its mRNA increased up to 90.1% in Put-treated cells and up to 86.4% in Put-treated Cis-incubated cells. The ∆9 splice variant was expressed at a minor level and remained almost unchanged in Put- and/or Cis-treated cells. The results of this experiment indicate that the prevention of CD4^+^ T cell death in response to Put is associated with the induction of alternative splicing of *RAD51A* pre-mRNA, leading to the dominant expression of FL *RAD51*.

### 2.6. Induction of the Full-Length RAD51A Splice Variant with Splice-Switching Oligonucleotides Leads to the Protection of CD4^+^ T Cells against Cisplatin

**Figure 6:**
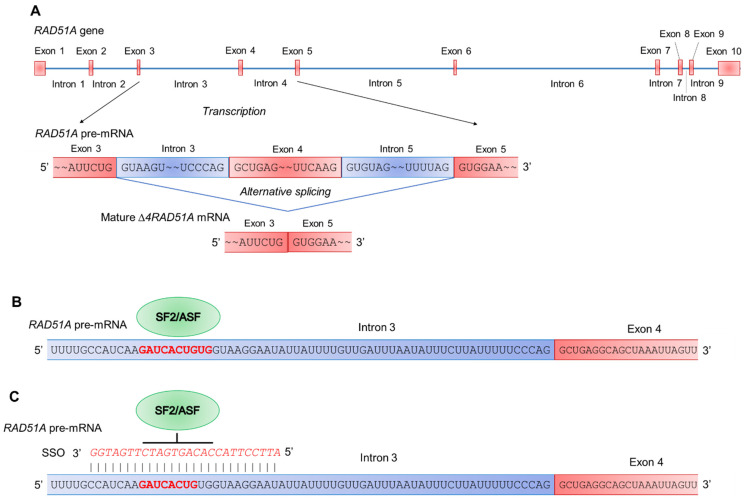
Schematic presentation of *RAD51A* alternative splicing. (**A**) Deletion of exon 4 as a result of alternative splicing and maturation of ∆*4RAD51A* mRNA. (**B**) Splicing regulator proteins SF2/ASF (shown as a green ellipse) interact with its binding sites (shown in bold red font) within intron 3 of RAD51A pre-mRNA. (**C**) Cell transfection with the 26-mer-specific antisense SSO for *RAD51A* (presented in red italic font) blocks the SF2/ASF proteins from binding to their binding sites. Exons are shown as red boxes, and introns are shown as blue boxes.

To further examine the involvement of *RAD51A* alternative splicing in the prevention of Cis-induced cell death, we modulated it with SSO, which can switch alternative splicing toward the FL variant (Figure 6). CD4^+^ T lymphocytes were transfected with a 26-mer oligonucleotide complementary to *RAD51* pre-mRNA and base pairing with the binding sites located in intron 3 of the splicing regulator protein SF2/ASF [29,30].

**Figure 7:**
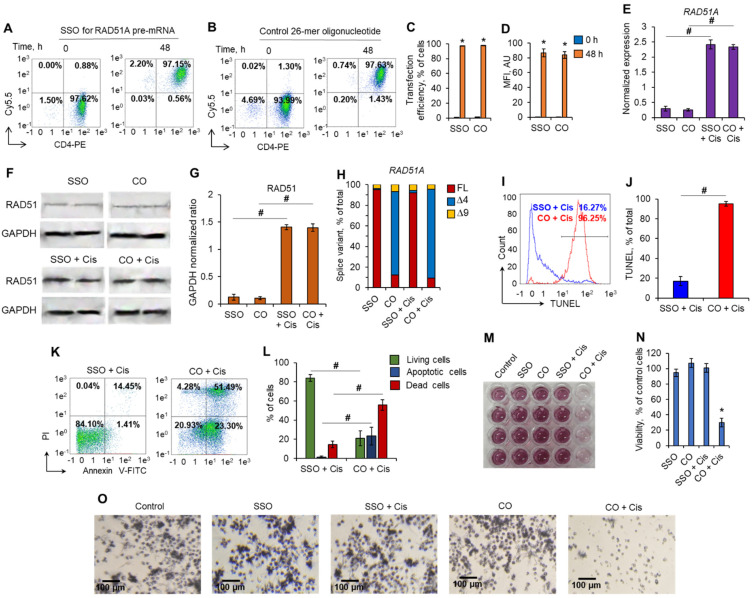
Modulation of *RAD51A* alternative splicing results in CD4^+^ T cell protection against cisplatin-induced apoptosis. Transfection efficiency of CD4^+^ T cells. Representative flow cytometry plots of cells transfected with Cy5.5-labeled (**A**) SSO for RAD51A or (**B**) a control 26-mer oligonucleotide 72 h posttransfection. (**C**) Efficiency of transfection. (**D**) Mean fluorescence intensity (MFI) of Cy5.5-positive cells. (**E**) mRNA levels of RAD51A measured by real-time RT-PCR in transfected CD4^+^ T lymphocytes incubated with cisplatin. mRNA levels were normalized relative to the expression of the reference gene *18S*. (**F**) Western blotting for RAD51 protein and the reference protein GAPDH in transfected cells incubated with cisplatin. (**G**) Results of RAD51 protein quantification relative to GAPDH. (**H**) mRNA levels of *RAD51A* splice variants measured by real-time RT-PCR in CD4^+^ T cells. (**I**) Representative TUNEL flow cytometry diagrams for cells transfected with oligonucleotides and incubated with cisplatin. (**J**) Results of TUNEL assay for flow cytometry. (**K**) Representative flow cytometry plots for cells labeled with annexin V-FITC and propidium iodide after transfection with oligonucleotides and incubation with cisplatin. The proportions of live cells (low left quadrants), apoptotic cells (low right quadrants), and dead cells (two upper quadrants) are presented. (**L**) Histograms of live, apoptotic, and dead cells measured by flow cytometry. (**M**) Representative photo of the MTT test for transfected CD4^+^ T lymphocytes incubated with cisplatin. (**N**) Results of MTT test quantification. (**O**) Bright-field optical images of the MTT test for transfected CD4^+^ T cells exposed to Cis. AU: arbitrary units; Cis: cisplatin; CO: control nonspecific oligonucleotide; FL: full-length splice variant. SSO: splice-switching oligonucleotide. ∆4: mRNA splice variant with the deletion of exon 4. ∆9: mRNA splice variant with the deletion of exon 9. *n* = 4. * *p* ≤ 0.05 vs. initial nontransfected cells. # *p* ≤ 0.05.

Forty-eight hours post-transfection, the transfection efficiency for both SSO and control nonspecific oligonucleotides was 97.01–98.4% (Figure 7A–D). Transfected cells were incubated with 0.5 µM of Cis. The mRNA levels of RAD51A and its splice variants were measured in the transfected cells incubated with Cis. Real-time RT-PCR revealed a more than fivefold increase in total *RAD51* expression in Cis-incubated cells transfected with all nucleotides (Figure 7E). Western blotting results of total RAD51 protein were in agreement with the results from real-time RT-PCR (Figure 7F,G). We observed the induction of RAD51 protein levels in transfected cells in response to treatment with Cis. Transfection of cells with SSO to *RAD51A* pre-mRNA resulted in significant induction of the FL variant and downregulation of the ∆4 splice variant in both Cis-treated and nontreated cells (Figure 7H). Control nonspecific oligonucleotides were not able to modulate alternative splicing or to induce FL variants. The mRNA level of the ∆9 splice variant remained unchanged in transfected cells regardless of incubation with Cis. This experiment demonstrated that the greatest contribution to the increase in total RAD51A in SSO-transfected cells is made by an increase in the amount of FL splice variant, and the predominance of FL variant is saved for Cis-treated cells. Next, we investigated the influence of SSO transfection on cell survival. Induction of *RAD51A* alternative splicing with SSO significantly decreased the proportion of cells having DNA damage induced by Cis, which was detected by TUNEL assay and flow cytometry (Figure 7I,J). Cis-treated cells transfected with control oligonucleotides did not prevent DNA damage in cells. Transfection of cells with SSO led to the prevention of apoptotic cell death. A total of 79.2–85.9% of SSO-transfected cells were alive after Cis treatment, while only 16.4–30.6% of cells transfected with control 26-mer oligonucleotides remained alive (Figure 7K,L). This observation was in accordance with the results of the MTT test, which showed that SSO-transfected cells were more resistant to Cis (Figure 7M–O). The viability of cells transfected with SSO and treated with Cis did not differ from that of nontransfected cells, while Cis treatment of cells transfected with control oligonucleotides resulted in a decrease in viability up to 24.2–35.6%. The results of this experiment demonstrated that the induction of the alternative splicing of *RAD51A* pre-mRNA toward the FL variant using antisense SSO results in the protection of cells against DNA damage and cell death.

## 3. Discussion

Most genes of higher eukaryotes have interrupted structures at which coding regions, exons, alternate with noncoding sequences, introns. Gene transcription leads to the formation of pre-mRNA, a molecule that has both exons and introns. During the maturation of mRNA, the splicing of exons and excision of introns by spliceosomes occurs [31]. Posttranscriptional maturation of pre-mRNA plays an essential role in providing biodiversity of protein products encoded by a single gene due to the process of alternative splicing of pre-mRNA. In this process, particular exons, or parts of exons, may be included within or excluded from the final mature mRNA. Consequently, the proteins translated from alternatively spliced pre-mRNAs will contain differences in their amino acid sequence and, often, in their biological functions [32]. An exon may be constitutive (always included in the mRNA) or alternative (may be included or excluded) to generate alternative splice variants. The usage of a splice site may be enhanced or suppressed by its proximity to local cis-regulatory sequences such as exonic splicing enhancers or silencers and intronic splicing enhancers or silencers [33,34]. The cis-regulatory sequences are in turn bound by *trans*-acting factors or splicing factors. Most often, they are serine/arginine-rich proteins (SR proteins). The spliceosome deletes intron and exon ligation during splicing, while the functioning of SR proteins is crucial for the determination of the sites that will be deleted or retained.

This study, for the first time, demonstrated a link between PAs, the alternative splicing of *RAD51A* pre-mRNA, and cell protection against cell death due to DNA damage. PAs were able to protect normal activated CD4^+^ T lymphocytes but not Jurkat or K562 cancer cell lines due to the action of Dox, Cis, or Irt (Figure 1). These cytotoxic compounds are widely used anticancer chemotherapeutics targeting DNA replication and are able to induce double-strain DNA damage by different mechanisms [35,36,37,38,39,40]. Normal CD4^+^ T cells were more sensitive to cytotoxic compounds than cancer cells, as demonstrated by the MTT test (Figure 1), TUNEL assay (Figure 2), and the measurement of live, apoptotic, and dead cells by flow cytometry (Figure 2). However, pretreatment of lymphocytes with each PA resulted in a significant reduction in cells with DNA damage, which increased their survival and viability. All three PAs did not show such an effect on Jurkat and K562 cancer cells and were not able to decrease the rate of DNA damage or increase cell viability. The ability of PAs to reduce oxidative stress and DNA damage is rather well studied [21,41,42], while the precise mechanisms of DNA protection by PAs remain to be determined. One of the early discoveries about PA–DNA interactions was the observation that PAs could stabilize double-stranded DNA due to charge neutralization [43] and by docking into the major or minor grooves [44]. Other researchers directly studied the effect of PA–DNA interactions on double-strand integrity from a structural standpoint [45,46]. Later, the promotion of homology-directed DNA repair by PAs due to the activation of RAD51 was described [11]. Our current work belongs to the latter type, where we showed the induction of RAD51A expression in response to DNA damage in cancer and normal cells (Figure 4). However, unlike the above-mentioned study, we demonstrated that PAs can induce alternative splicing of RAD51A pre-mRNA only in normal CD4^+^ T cells (Figure 5), and the shift of the pool of RAD51A mRNA from the predominant ∆4 splice variant toward the FL variant is associated with cell protection. The *RAD51A* gene consists of 10 exons that are subjected to alternative splicing during mRNA maturation (Figure 6A). The most abundant slice variants among murine and human cells are FL, ∆4, and ∆9 variants [47,48]. Exon 4 encodes the helix-turn-helix region of the protein. Its deletion (i.e., ∆4 splice variant) results in the loss of the ability to interact with BRCA1 and BRCA2, which may be important for the cellular response to DNA damage. BRCA2 has been shown to regulate both the intracellular localization and DNA-binding ability of this protein [49]. Exon 9 encodes a part of the so-called L2 region loop of the RAD51 protein, and its deletion (i.e., ∆9 splice variant) abrogates the DNA-binding capacity of the protein [50]. Dominant negative mutations in RAD51 protein variants are suggested to be associated with the Fanconi anemia subtype [51]. Pre-mRNAs of different members of *RAD51* are subjected to alternative splicing and are believed to have impacts on cancer progression [52,53,54]. Our results correspond to the inability of truncated RAD51A splice variants to promote homological recombination and reduce DNA damage because Put induced the predominance of the FL variant and prevented the death of normal CD4^+^ T cells. Although the questions of why PAs did not induce RAD51A alternative splicing in cancer cells and the mechanism of its induction by PAs remain to be investigated, we demonstrated the involvement of alternative splicing of RAD51A pre-mRNA in cell protection against DNA damage. The modulation of alternative splicing was performed using SSO targeting pre-mRNA of *RAD51A* at the cis-regulatory sequences 5′-GAUCACUG-3′, which is the binding site for *trans*-regulatory SR protein SF2/ASF [55] (Figure 6B,C). The steric block of such a powerful splicing factor resulted in the predominant expression of the FL splice variant in Cis-treated CD4^+^ T cells (Figure 7). The shift of the splicing patterns (Figure 7H) was associated with a decrease in DNA damage (Figure 7I,J) and cell protection against Cis-induced apoptosis (Figure 7K–O). The results of this work suggest that the cytoprotective properties of PAs are associated with alternative splicing of *RAD51A* pre-mRNA in normal human CD4^+^ T lymphocytes but not in cancer cells. The difference in the sensitivity between normal and cancer cells for PAs may become the basis for the use of these compounds to protect normal lymphocytes during lymphoblastic chemotherapy.

A number of studies demonstrated the role of RAD51 in cancer progression and the levels of the RAD51 protein are elevated in many cancer cell lines and in primary tumors [56]. RAD51 overexpression can result in improper and hyper-recombination, namely contributing to genomic instability and genetic diversity [57,58,59,60]. These, in turn, might drive regular cells towards neoplastic transformation or further contribute to cancer progression. Considering a central role or RAD51 in homological recombination during DNA, it is only logical that its activity is regulated by a set of partner proteins and modulators [5]. We believe that AS of RAD51A represents a mechanism of fine-tuning of DNA repair at the level of modulation of partner protein interaction with RAD51A splice variants. This conclusion must be supported in additional study and the exact role of RAD51A splice variants in cancer progression remains to be determined. However, RAD51 involvement in cell survival in genotoxic conditions and its different abilities for AS in cancer and normal cells makes it a promising target for anticancer applications.

Although most PA-related anticancer strategies are aimed to decrease PAs concentration in tumor cells or tumor microenvironment [61,62], some cell types including leukemia cells remain anergic for PAs at low doses. This promising fact, in case of further study, makes possible the clinical evaluations of polyamines administration in combination with standard chemotherapy.

In conclusion, in our work, we demonstrated for the first time that the induction of alternative splicing of *RAD51A* pre-mRNA toward FL variant by PAs results in the protection of normal human CD4^+^ T cells against DNA damage. Consequently, PAs can be used for the protection of normal immune cells during DNA-targeting chemotherapy. Unfortunately, the results of this work do not answer the question about the mechanism by which PAs induce alternative splicing of *RAD51A* pre-mRNA and why this process is abolished in cancer cells. Therefore, future st udies are necessary to resolve these issues. However, this descriptive study demonstrated the impact of PAs on the alternative splicing of *RAD51A* pre-mRNA and demonstrated its role in cell protection against DNA-damage-associated cell death.

## 4. Materials and Methods

### 4.1. Cell Purification and Cultivation

The study was approved by the Ethical Committee of the Institute of Biomedical Chemistry; written informed consent was obtained from all participants. Blood from healthy 18–25-year-old donors (*n* = 4) was collected in Vacuette K3EDTA tubes (Greiner Bio-One, Kremsmünster, Austria). Peripheral blood mononuclear cells (PBMCs) were isolated using Lympholite-H (Cedarlane, Burlington, ON, Canada) density gradient centrifugation. CD4^+^ T cells were purified from PBMCs using a CD4^+^ Human Isolation Kit (Miltenyi Biotec, Bergisch Gladbach, Germany) according to the manufacturer’s instructions. The purified cells were cultured according to a previously described protocol [63,64]. Briefly, CD4^+^ T cells were seeded at 5 × 10^5^ cell/mL and cultured in 25 cm^2^ flasks in RPMI 1640 cell medium (Thermo Fisher Scientific Inc., Waltham, MA, USA) supplemented with 10% FBS (fetal serum bovine, Capricorn Scientific, Ebsdorfergrund, Germany), with 5 µg/mL anti-CD28 (eBioscience Inc., San Diego, CA, USA), 5 µg/mL anti-CD3 mAbs (MedBioSpectr, Moscow, Russia), and 100 U/mL rHu IL-2 (R&D Systems, Minneapolis, MN, USA). Cells were cultivated in 5% CO_2_/95% air in a humidified atmosphere at 37 °C and restimulated every three days with complete medium supplemented with IL-2 and anti-CD3 and anti-CD28 antibodies.

Acute T cell leukemia Jurkat cells and chronic myeloid leukemia K562 cells (both from ATCC, Manassas, VA, USA) were grown in RPMI-1640 supplemented with 5% fetal bovine serum (Capricorn Scientific, Ebsdorfergrund, Germany) and 1% sodium pyruvate (Paneco, Moscow, Russia), and cells were grown in 5% CO_2_/95% air in a humidified atmosphere at 37 °C. Cell lines were tested for mycoplasma contamination before the experiment using the Mycoplasma Detection Kit PlasmoTest™ (InvivoGen, San Diego, CA, USA).

### 4.2. A. Poptosis Induction and Toxicity Assays

To induce genotoxicity, Jurkat, K562 or normal CD4^+^ T lymphocytes were incubated for 72 h in 96-well plates (TPP, Trasadingen, Switzerland) with 1 µM doxorubicin (Dox, Veropharm, Moscow, Russia), 0.5 µM cisplatin (Cis, cis-diamminine-dichloroplatinum(II), Sigma–Aldrich, St. Louis, MO, USA), or 1 µM irinotecan (Irt, Veropharm, Moscow, Russia) in the presence of 10 µM of each of three PAs: Spm (spermine tetrahydrochloride), Spd (spermidine trihydrochloride), or Put (putrescine dihydrochloride, all from Sigma–Aldrich, St. Louis, MO, USA). The concentrations of PAs and genotoxic drugs were established in preliminary experiments (Figure S1A–C in the Supplementary File). Cell viability was tested by measuring the conversion of the tetrazolium salt 3-(4,5-dimethyl-thiazol-2-yl)-2,5-diphenyltetrazolium bromide (Serva, Heidelberg, Germany) to formazan (MTT test). IC50 and IC90 values (the concentration of the enzyme where the response is reduced by 50% and 90%, respectively) were calculated from curve-fitting equations [65]. Bright-field optical images were acquired using a Leica DMI300 inverted microscope (Leica Microsystems, Wetzlar, Germany).

To measure apoptosis, incubated cells were resuspended in PBS (Paneco, Moscow, Russia) and incubated with annexin V-FITC and propidium iodide (PI) from a FITC Annexin V/Dead Cell Apoptosis Kit (Life Technologies, Carlsbad, CA, USA) according to the manufacturer’s protocol. The counting of 5 × 10^4^ cells at each point was performed by flow cytometry with a MACS Quant Analyzer 10 (Miltenyi Biotec, Bergisch Gladbach, Germany) as we previously described [66].

The proportion of cells with DNA damage was estimated by the terminal deoxynucleotidyl transferase-mediated d-UTP nick end labeling (TUNEL) assay [67,68] using the FlowTACS Apoptosis Detection Kit (R&D Systems, Minneapolis, MN, USA) according to the manufacturer’s protocol and flow cytometry.

### 4.3. Cell Transfection with Splice-Switching Oligonucleotide

The transfection of CD4^+^ T lymphocytes with 26-mer splice-switching oligonucleotide (SSO) base pairing with RAD51A pre-mRNA or control 26-mer oligonucleotide was performed using Lipofectamine 2000 (Invitrogen, Grand Island, NY, USA) according to the manufacturer’s protocol. The nucleotides (custom synthesized by Evrogen, Moscow, Russia) were uniformly modified with 2′-O-(2-methoxy) ethyl sugars (2′MOE), a phosphorothioate backbone, and 5′-methyl cytosine as described in [69] and conjugated with Cy5.5 dye. The SSO sequence is provided in Table 1 and Figure 6B. A BLAST search for SSO target sequences revealed no other perfect sequence matches within the human genome. To determine the efficiency of transfection, the cells were labeled with CD4-phycoerythrin (PE) antibodies (Miltenyi Biotec, Bergisch Gladbach, Germany) according to the manufacturer’s protocol, and Cy5.5-positive cells were counted by flow cytometry with a MACS Quant Analyzer 10 (Miltenyi Biotec, Bergisch Gladbach, Germany). The cellular load of the SSO or control oligonucleotide was determined by the mean fluorescence intensity (MFI) of Cy5.5-positive cells.

### 4.4. RNA Isolation and Real-Time RT-PCR

A previously described protocol was followed [70]. Briefly, total RNA from cells were extracted using a PureLink RNA Mini Kit (Life Technologies, Carlsbad, CA, USA). Five micrograms of total RNA was reverse-transcribed using the MMLV RT Kit (Evrogen, Moscow, Russia) in a 25 mL reaction mixture, followed by real-time RT-PCR using DTprime5 (DNA Technology, Protvino, Russia). The reaction mix was prepared using qPCR mix-HS SYBR (Evrogen, Moscow, Russia) according to manufacturer recommendations using the primers listed in Table S1 in the Supplementary File. Two-temperature annealing/extension cycles were used. The fluorescence was measured at the end of the annealing step. Melting curve analyses were performed at the end of the reaction (after the 35th cycle) between 60 °C and 95 °C to assess the quality of the final PCR products. The threshold cycles and C(t) values were calculated by fixing the basal fluorescence at 300 units. The standard curve of the reaction effectiveness was performed using serially diluted mixtures (1:40, 1:80, 1:160, 1:320, and 1:640) of all experimental cDNA samples in duplicate for each gene and *18S* RNA separately. Calculation of the relative RNA concentration was performed using DTPrime5 software. Data were presented as ratios of mRNA/*18S* mRNA.

### 4.5. Western Blotting

Cells were lysed in 1 mL of TBE buffer (89 mM Tris, 89 mM H_3_BO_3_, 2 mM EDTA, pH = 8.0) by ultrasonic disruption (50 W, 2 min) using a Sonic Dismembrator (Thermo Fisher Scientific Inc., Waltham, MA, USA). Cell lysates were centrifuged for 10 min at 12,000× *g* to remove cell debris. Protein in samples was measured using the Bradford protein assay (Pierce Biotechnology, Rockford, IL, USA). Bovine serum albumin was used for serial dilutions for the calibration curve. The total protein extracted from cells (50 μg of total protein) was dissolved in 50 mM Tris-HCl at a pH of 6.8, 1% sodium dodecyl sulfate, 2 mM EDTA, 1% 2-mercaptoethanol, and 7.5% glycerol and denatured by heating at 100 °C for 10 min. Proteins were separated in gradient PAAG [71] (100 V; 2 h) using NuPAGE^®^ Novex^®^ 4–12% Bis-Tris Protein Gels (Life Technologies, Carlsbad, CA, USA). Proteins were transferred onto the nitrocellulose membrane in Novex transferring buffer (Invitrogen, Grand Island, NY, USA) at 40 V for 3 h. The membranes were stained with Ponceau S (Sigma–Aldrich, St. Louis, MO, USA) [72]. After soaking in the blocking solution Blotting-Grade Blocker (Bio–Rad, Hercules, CA, USA), the membranes were incubated with monoclonal antibodies to glyceraldehyde-3-phosphate dehydrogenase (anti-GAPDH) or anti-RAD51 (both from Abcam, Cambridge, MA, USA) diluted to 1:1000. Membranes were washed in Tris-buffered saline at a pH of 7.6, with 0.1% Tween-20 (Invitrogen, Grand Island, NY, USA) and incubated with secondary antibodies conjugated with horseradish peroxidase (Cell Signaling, Danvers, MA, USA). Membranes were visualized using a Super Signal chemiluminescent kit (Pierce Biotechnology) and documented in a ChemiDoc^TM^ XRS imaging system (Bio–Rad, Hercules, CA, USA). Relative amounts of proteins were determined by densitometry in GelAnalyzer 19.1 (www.gelanalyzer.com, accessed on 17 January 2022).

### 4.6. Statistics

Statistical analysis was performed with 2-way ANOVA and Student’s *t*-test using SPSS 25 software (IBM SPSS Statistics, Armonk, NY, USA). Bonferroni modification of Student’s *t*-test was applied as appropriate. The results are expressed as the mean ± standard error of the mean (SEM). *p* ≤ 0.05 was considered significant.

## Figures and Tables

**Table 1:** Oligonucleotides used for CD4^+^ T cell transfection.

Target	Sequence (5′-3′)
SSO for *RAD51A* pre-mRNA	ATTCCTTACCACAGTGATCTTGATGG
Control 26-mer oligonucleotide	AUGUGCCGUAGGUGAGGCCUCACGUU

## Data Availability

The data presented in this study are available on request from the corresponding author. The data are not publicly available due to ethical restrictions.

## Supplementary Materials

The following supporting information can be downloaded at: https://www.mdpi.com/article/10.3390/ijms23031863/s1.
