# Supplementary material for: Cytoprotective Activity of Polyamines Is Associated with the Alternative Splicing of RAD51A Pre-mRNA in Normal Human CD4+ T Lymphocytes
Source: Int J Mol Sci. 2022 Feb 7;23(3):1863. doi: 10.3390/ijms23031863 (PMC8837172; doi:10.3390/ijms23031863)
Supplement: Supplementary file 1 [file ijms-23-01863-s001.zip › ijms-1581246-supplementary.pdf]

Cytoprotective Activity of Polyamines is Associated with the Alternative Splicing of RAD51A Pre-mRNA in Normal Human CD4+ T Lymphocytes

Yulia A. Gladilina[1], Lylia Bey[2], Abdullah Hilal[1], Ekaterina V. Neborak[2], Varvara G. Blinova[1], Dmitry D. Zhdanov[1,2,*]

[1] Institute of Biomedical Chemistry, Pogodinskaya st. 10/8, Moscow, 119121, Russia; leonova_y@mail.ru (Y.A.G.); hilalabdullahh@gmail.com (A.H.); varya.blinova@list.ru (V.G.B.)

[2] Department of Biochemistry, Peoples' Friendship University of Russia (RUDN University), Miklukho—Maklaya Street 6, Moscow, 117198, Russia; beylylia60@gmail.com (L.B.); neborak-ev@rudn.ru (E.V.N)

Correspondence: zhdanovdd@gmail.com

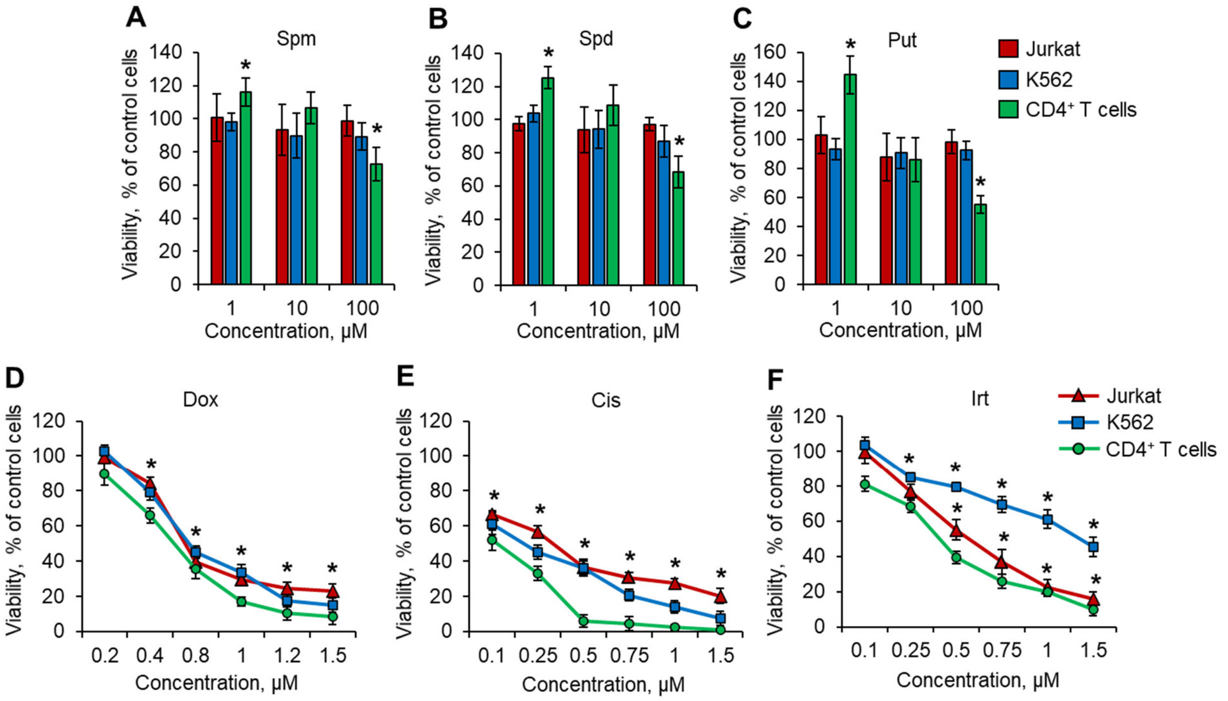

Figure S1. Results of the MTT test for cancer Jurkat and K562 cells and normal human CD4+ T lymphocytes incubated with different concentrations of (A) spermine (Spm), (B) spermidine (Spd), (C) putrescin (Put), (D) doxorubicin (Dox), (E) cisplatin (Cis), or (F) irinotecan (Irt) for 48 h. N = 8. * $p \leq 0.05$ vs. control nontreated cells.

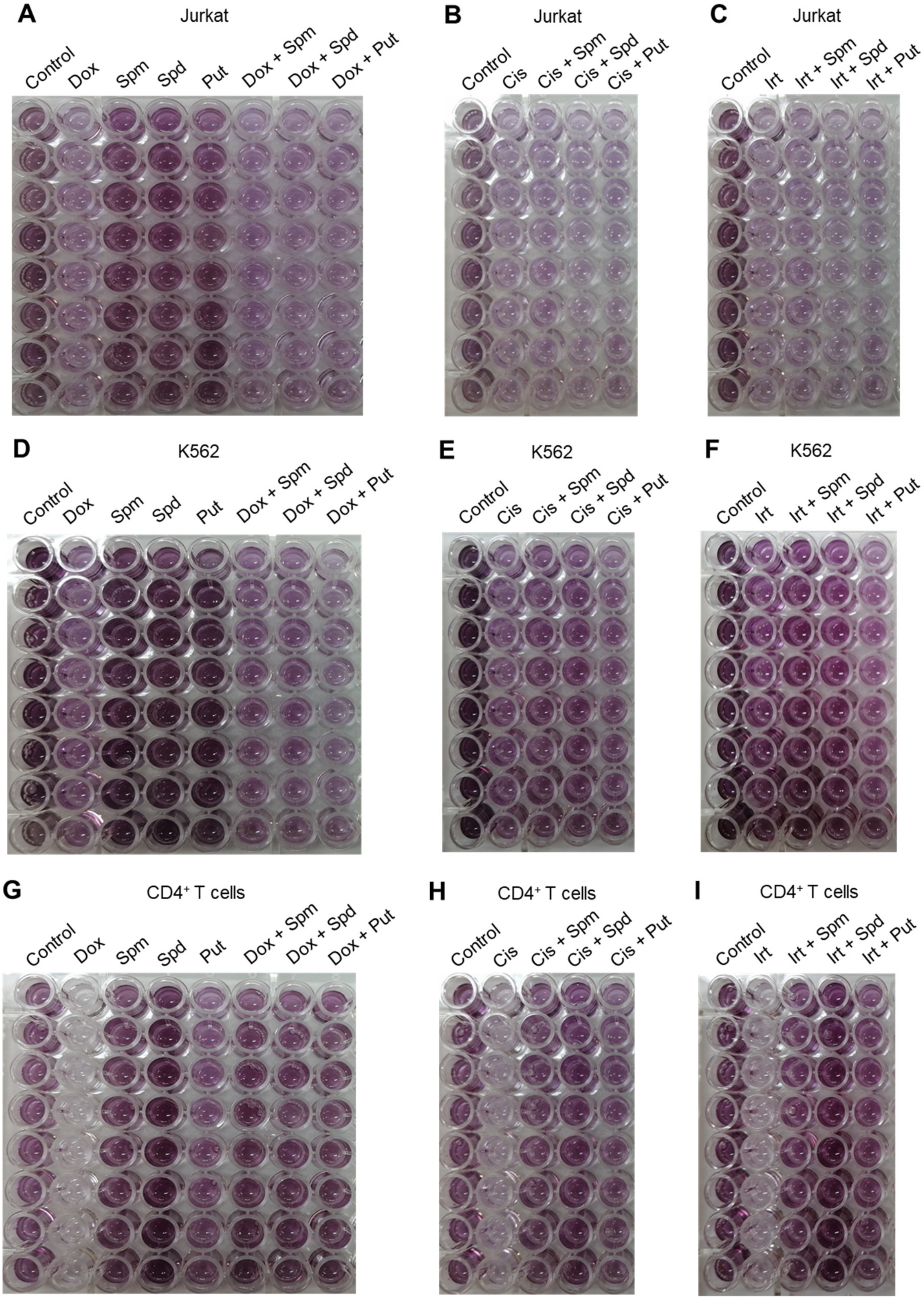

Figure S2. Representative photos of the MTT test for cells incubated with 10 μM of each PA for 72 h. The results of the MTT test for cancer (A – C) Jurkat, (D – E) K562 or (G – I) normal $CD4^+$ T cells incubated with genotoxic agents: 1 μM doxorubicin (Dox), 0.5 μM cisplatin (Cis) or 1 μM irinotecan (Irt) in the presence of 10 μM or in the absence of each PA. Spd, spermidine; Spm, spermine; Put, putrescine.

Table S1. List of primers used for real-time RT-PCR.

Target	Sense 5' – 3'	Antisense 5' – 3'	Product size, bp.	Tann, °C
RAD51A Total	ggggcaagcgagtagagaag	gctgcatctgcattgccatt	151	60
FL RAD51A	ctacttcaaggtggaattgag	ctgctacaccaaactcatcagc	454	57
RAD51A Δ4	gataaaattctggtggaattgag	ctgctacaccaaactcatcagc	456	58
RAD51A Δ9	gccatgatggtagaatctag	ctcagatacaatctcatcagc	162	60
RAD51B	gtgtggcttgtggatccctc	ctgggaaaacgggattctgc	196	61
RAD51C	cgctgtcgtgactacacaga	ggctgatcatttgctgggct	181	61
RAD51D	tccagatgctggatgtgctg	ctgctgacctcccagaagtg	134	62
18S	ggatccattggagggcaagt	acgagctttttaactgcagcaa	91	62
